# miR-363-5p regulates endothelial cell properties and their communication with hematopoietic precursor cells

**DOI:** 10.1186/1756-8722-6-87

**Published:** 2013-11-21

**Authors:** Ana Costa, Joana Afonso, Catarina Osório, Ana L Gomes, Francisco Caiado, Joana Valente, Sandra I Aguiar, Francisco Pinto, Mário Ramirez, Sérgio Dias

**Affiliations:** 1 Angiogenesis Laboratory, Centro de Investigação em Patobiologia Molecular (CIPM), Instituto Português de Oncologia Francisco Gentil de Lisboa, EPE. Rua Professor Lima Basto, Lisbon 1099-023, Portugal; 2 Neoangiogenesis Group, Instituto Gulbenkian de Ciência, Oeiras, Rua da Quinta Grande, 6, Oeiras 2780-156, Portugal; 3 Instituto de Medicina Molecular, Faculdade de Medicina da Universidade de Lisboa, Edificio Egas Moniz, Av. Prof Egas Moniz, Lisbon 1649-028, Portugal; 4 Cardiff School of Biosciences, Biomedical Sciences Building, Museum Avenue, Biomedical Sciences Building, Museum Avenue, Cardiff, UK

**Keywords:** Bone marrow, Endothelial cell, Hematopoietic progenitors, miRNA, Cell interactions

## Abstract

Recent findings have shown that the blood vessels of different organs exert an active role in regulating organ function. In detail, the endothelium that aligns the vasculature of most organs is fundamental in maintaining organ homeostasis and in promoting organ recovery following injury. Mechanistically, endothelial cells (EC) of tissues such as the liver, lungs or the bone marrow (BM) have been shown to produce “angiocrine” factors that promote organ recovery and restore normal organ function. Controlled production of angiocrine factors following organ injury is therefore essential to promote organ regeneration and to restore organ function. However, the molecular mechanisms underlying the coordinated production and function of such “angiocrine” factors are largely undisclosed and were the subject of the present study. In detail, we identified for the first time a microRNA (miRNA) expressed by BM EC that regulates the expression of angiocrine genes involved in BM recovery following irradiation. Using a microarray-based approach, we identified several miRNA expressed by irradiated BMEC. After validating the variations in miRNA expression by semi-quantitative PCR, we chose to study further the ones showing consistent variations between experiments, and those predicted to regulate (directly or indirectly) angiogenic and angiocrine factors. Of the mi-RNA that were chosen, miR-363-5p (previously termed miR-363*) was subsequently shown to modulate the expression of numerous EC-specific genes including some angiocrine factors. By luciferase reporter assays, miR-363-5p is shown to regulate the expression of angiocrine factors tissue inhibitor of metalloproteinases-1 (Timp-1) and thrombospondin 3 (THBS3) at post-transcriptional level. Moreover, miR-363-5p reduction using anti-miR is shown to affect EC angiogenic properties (such as the response to angiogenic factors stimulation) and the interaction between EC and hematopoietic precursors (particularly relevant in a BM setting). miR-363-5p reduction resulted in a significant decrease in EC tube formation on matrigel, but increased hematopoietic precursor cells adhesion onto EC, a mechanism that is shown to involve kit ligand-mediated cell adhesion. Taken together, we have identified a miRNA induced by irradiation that regulates angiocrine factors expression on EC and as such modulates EC properties. Further studies on the importance of miR-363-5p on normal BM function and in disease are warranted.

## Findings

### Background

The bone marrow (BM) microenvironment consists of different cell types, grouped in “niches”, defined according to the cellular composition and also the signals produced [1,2]. Detailed knowledge of the regulation and composition of the BM niches, including the osteoblastic and the vascular niches, is essential for our understanding of BM function and may also contribute towards the discovery of therapeutic targets to treat BM diseases. Emerging evidence suggests the “vascular niche”, and bone marrow endothelial cells (BMEC) in particular, conveys signals to hematopoietic progenitor and stem cells, promoting BM recovery via instructive “angiocrine” signals that tightly regulate the hematopoietic differentiation process [3,4]. The coordinated production and release (in such cases) of instructive signals is crucial for adequate BM recovery and function; hematopoietic differentiation and exit into peripheral organs is tightly regulated by the instructive signals from the BM vascular niche [3,5]. In particular, the communication between BMEC and hematopoietic elements, namely the hematopoietic stem and progenitor cells, is crucial for normal BM function and for maintaining BM integrity following stress. Whole body irradiation has been used to study BM turnover, since it rapidly induces BM cells apoptosis and allows detailed study of the mechanisms involved in BM cell recovery [6,7]. Whole body irradiation is also clinically relevant as a majority of cancer patients, most notably those suffering from hematological malignancies, receive some form of radiotherapy [8,9]. Little is known about the role of miRNAs in the BMEC that contribute to regulate the BM function and recovery. miRNAs are recognized post-transcriptional regulators of gene expression through mRNA targeting and/or translational repression thereby modulating biological homeostasis [10]. In the present study, we discovered that miR-363-5p (previously termed miR-363*) is expressed by EC and is induced by irradiation *in vivo* and *in vitro.* miR-363-5p regulates the expression of angiocrine factors in EC, affects EC angiogenic properties and also modulates the interaction between EC and hematopoietic cells.

### Results

#### miRNA expression profile of irradiated whole BM is partially mirrored on irradiated isolated BMEC

**Figure 1:**
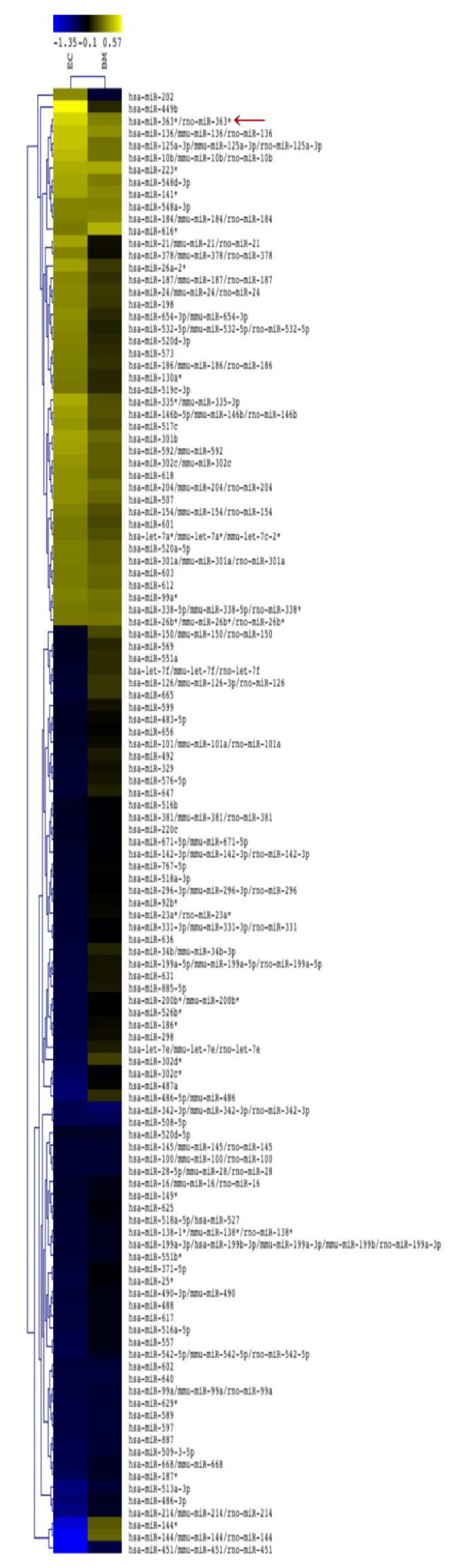
**miRNA profiling in bone marrow and bone marrow endothelial cells.** miRNAs differentially expressed (120) in irradiated BM and BMEC. Only expression values with p < 0.05 were considered. Unsupervised average linkage hierarchical clustering (Euclidean distance) was performed. The color display encodes the log2 of the expression changes, where varying shades of yellow and blue indicate up and down regulation, respectively.

**Figure 2:**
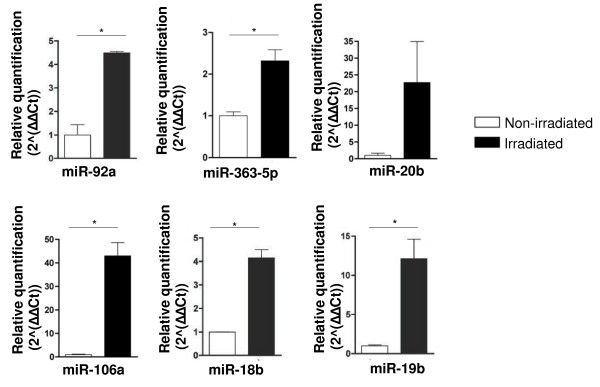
**Expression of miR-363-5p cluster in irradiated HUVECs.** Expression of the five miRNAs clustered with miR-363-5p in irradiated compared to non-irradiated endothelial cells, showing an increase of clustered miRNAs 24 h post-irradiation. Error bars represent s.e.m. of the normalized mean expression of irradiated relative to non-irradiated endothelial cells. * P ≤ 0.05 by Student’s t test.

miRNAs were identified on BMEC and on whole BM after irradiation; we identified 120 miRNAs whose expression showed similar variations between irradiated whole BM and BMEC, suggesting these would be mainly expressed by the latter (Figure 1). miRNA expression from the microarrays was validated by qRT-PCR on BMEC and on whole BM samples; we validated the expression of five miRNAs (Additional files 1 and 2). From the miRNAs selected, miR-363-5p showed the most consistent level increase considering both microarrays and validation by qRT-PCR (Figure 1 and Additional file 2). To confirm that miR-363-5p was induced upon irradiation and to establish a suitable *in vitro* system to manipulate, we irradiated primary endothelial cells (HUVEC). We observed a significant increase in miR-363-5p levels, 24 h after irradiation, by qRT-PCR, accompanied by the increase of expression of the five miRNAs belonging to the miR-363-5p cluster (miR-106a, miR-18b, miR-20b, miR-19b-2 and miR-92a-2) (Figure 2). Taken together, these results showed that miR-363-5p levels are increased upon irradiation *in vivo* and *in vitro*. Moreover, given that levels of miR-363-5p in resting HUVEC and BMEC are similar (Additional file 3), but the former are easier to transfect than the latter, we used HUVEC to perform miR-363-5p loss- and gain- of function experiments that technically require high number of cells. Having shown that miR-363-5p is up-regulated upon irradiation, we next investigated its role in the regulation of angiogenic and angiocrine-related genes and EC properties.

#### Angiogenic and angiocrine factors are regulated by miR-363-5p

**Figure 3:**
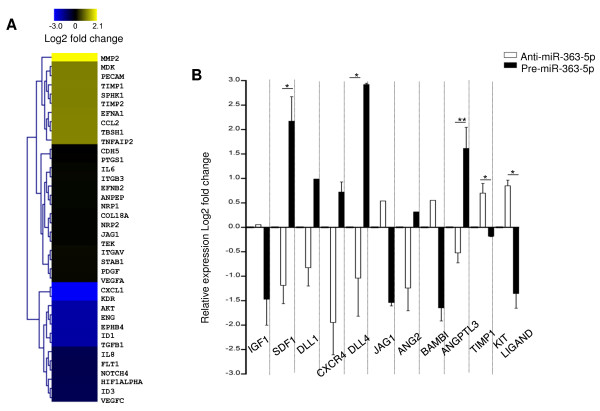
**Modulation of angiogenic and angiocrine genes by miR-363-5p. (A)** Expression profile of angiogenesis-related genes of EC with reduced level of miR-363-5p using pre-made PCR arrays. Unsupervised average linkage hierarchical clustering (Euclidean distance) was performed. The color display encodes the log2 of the expression changes normalized to scramble control, where varying shades of yellow and blue indicate up and down regulation, respectively. **(B)** Expression levels (log2 fold change) of angiocrine genes in endothelial cells having reduced or high levels of miR-363-5p compared to scramble control. Error bars represent s.e.m. of normalized expression mean. * P ≤ 0.05 ** P ≤ 0.01 by Student’s t test.

We investigated the angiogenic-related genes modulated by miR-363-5p on EC reducing the levels of miR-363-5p (anti-miR-363-5p transfected) and comparing it to scrambled-transfected controls. As transfection efficiency of HUVEC can vary (up to 90%, data not shown) the level of miR-363-5p, specifically the down-regulation achieved with anti-miR-363-5p, was confirmed by qRT-PCR (Additional file 4). We analyzed the gene expression profile of anti-miR-363-5p transfected EC versus scrambled controls, using commercially available pre-made PCR arrays for angiogenesis-related genes and also custom-made arrays for angiocrine factors as shown in Figures 3A and 3B, respectively. The gene expression profile analysis obtained from the angiogenesis-directed pre-made PCR arrays (Figure 3A) showed that EC with reduced levels of miR-363-5p had a significant decrease of expression of pro-angiogenic genes (e.g. ephrin B4 (EPHB4), endoglin, FLT1 (VEGFR-1), KDR (VEGFR-2), Notch 4, among others) and in turn, showed an increase of expression of anti-angiogenic genes (THBS1, TIMP1). The gene expression profile was validated by qRT-PCR (Additional file 5). Additionally, modulation of miR-363-5p in EC also affected the expression of angiocrine factors. As shown in Figure 3B, the miR-363-5p reduction led to a general decrease of the expression of angiocrine genes including stromal-derived factor 1 (SDF1), Delta ligand-like1, Delta ligand-like4, CXCR4, angiopoietin2 and angiopoietin-like3. In turn, an increase of expression of the angiocrine genes, kit ligand (stem cell factor), jagged-1 and TIMP1 was observed upon miR-363-5p reduction. Taken together, we show that levels of miR-363-5p in EC affected the expression of both angiogenic and angiocrine genes. However, we would like to clarify that we cannot, based solely on these data, conclude the modulated angiogenic and angiocrine genes are direct targets of miR-363-5p.

#### TIMP1 is a direct target of miR-363-5p

**Figure 4:**
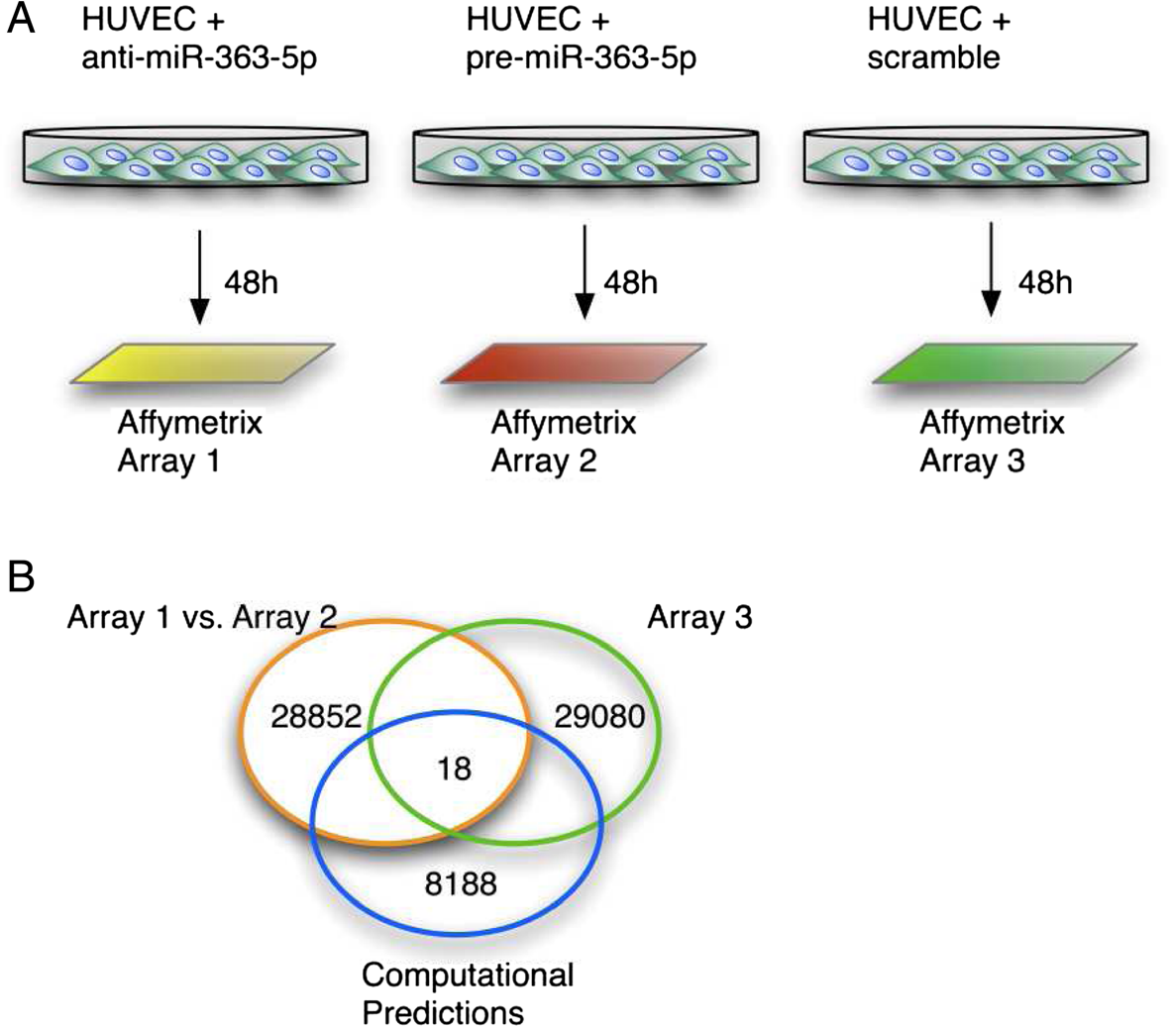
**Identification of putative direct targets of miR-363-5p. (A)** Schematic view of the strategy to find direct targets of miR-363-5p. **(B)** Venn diagram showing the direct putative target genes as the result of comparison of the arrays with computational predictions (miRBase).

Having defined candidate genes that appeared to be regulated by miR-363-5p, next we sought to define if some were under the direct regulation of miR-363-5p. To achieve this and as the identification of miRNAs targets can be complex, we followed a global approach to limit the search of direct targets of miR-363-5p and modulated EC with anti-miR-363-5p, pre-miR-363-5p or scramble control followed by transcriptome analysis using cDNA microarrays of human genome (Affymetrix GeneChip Human Gene 1.0 ST array) (Figure 4A). The comparison of the genes modulated upon miR-363-5p (high/low levels), and discarding unspecific variations (filtered by scramble control), in combination with the computational predictions obtained from miRBase resulted in the identification of putative 18 target genes, as shown in the Venn diagram (Figure 4B and Additional file 6). The known-pathways of the predicted target genes were investigated using the Ingenuity software, which allowed us to select genes with a known role in angiogenesis and/or in the modulation of angiocrine genes (the vascular niche). The expression of the genes identified from microarrays experiments was further validated by qRT-PCR (Additional file 7). We validated by qRT-PCR the expression of four angiocrine genes (TIMP1, SELE, IKBKG and THBS3), which were considered putative direct targets of miR-363-5p and thus were selected for further validation by Luciferase reporter assays. The regulatory 3’UTR of these four genes was cloned into pMIR-REPORT upstream the Luciferase gene and co-transfected into HUVEC with pre-miR-363-5p or scramble control. A specific and direct interaction between the miRNA and its target (wild-type UTR) can be assessed by the reduction of the luciferase reporter activity following transfection with pre-miR-363-5p. Conversely, if the miRNA binding site is abrogated (mutant UTR), the luciferase reporter should be unaffected following transfection with pre-miR-363-5p, as the miRNA is no longer able to recognize the mutated site. According to this approach, preliminary Luciferase assays using wild-type UTRs indicated that IKBKG and SELE might not be direct targets of miR-363-5p (results not shown), while THBS3 and TIMP1 are likely regulated by miR-363-5p.

**Figure 5:**
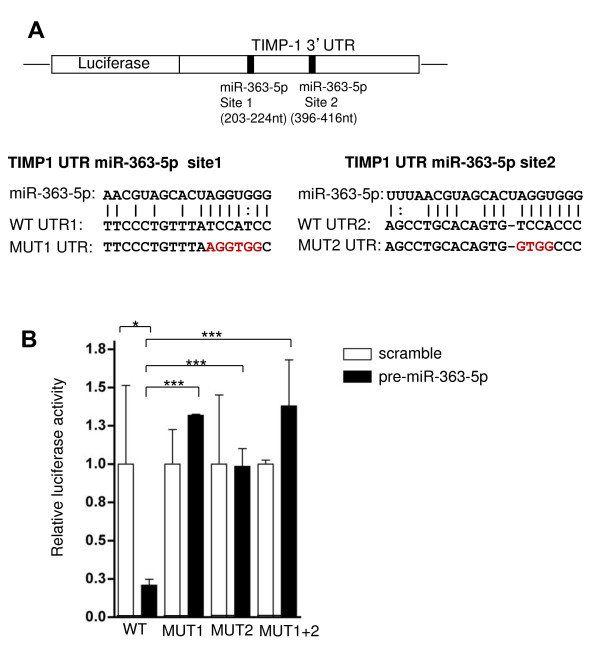
**miR-363-5p interaction with TIMP-1 UTR. (A)** Schematic view of the TIMP1 construct into pMIR-REPORT showing the two predicted binding sites in TIMP1 3’UTR. Sequence alignment of miR-363-5p and the 3’UTR TIMP1 is shown. Nucleotide mutations achieved by site-directed mutagenesis are colored in red. **(B)** Renilla luciferase activity normalized to Firefly activity of HUVEC 48 h post-transfection with pre-miR-363-5p relative to scramble control. Relative luciferase activity of wild-type (WT) 3’UTR constructs and mutation in the predicted site 1 (MUT1), site 2 (MUT2) and both sites mutated (MUT1 + 2) are shown. Errors bars are s.e.m. from three independent transfection experiments. * P ≤ 0.05, *** P ≤ 0.001 by Student’s t test.

TIMP1 is an inhibitor of matrix metallopeptidases involved in the degradation of the extracellular matrix. The 3’UTR of TIMP1 has two predicted binding sites for miR-363-5p (Figure 5A). The free energy of the hybrid miRNA:3’UTR for TIMP1 is −21.92 Kcal/mol and −15.15 Kcal/mol respectively for site1 and site2. The predicted energy of the hybrid for TIMP1 (site 1) is lower that −17 Kcal/mol, typical of specific interaction. To prove direct interaction of miR-363-5p and TIMP1 binding site, site-directed mutagenesis in the binding sites, specifically in 5 nucleotides of the seed sequence were performed. As a result, three mutant plasmids were generated comprising the mutagenesis of the first binding site (MUT1), the second binding-site (MUT2) or having both binding sites mutated (MUT1 + 2). As shown in Figure 5B, the increased levels of miR-363-5p lead to a drastic reduction of the Luciferase/Renilla activity in the wild-type 3’UTR, suggesting that miR-363-5p recognizes and interacts with the predicted binding sites. Conversely, this trend is abolished in all mutants, confirming the specificity of the interaction between miR-363-5p and TIMP1 3’UTR.

THBS3 contributes to extracellular structure and function [11], but it remains largely uncharacterized contrasting to thrombospondin-1 which well known for its anti-angiogenic properties. A reduction of Luciferase/Renilla activity was observed when miR-363-5p levels are augmented, trend that was reversed with THBS3 mutated 3’ UTR, showing that miR-363-5p regulates THBS3 (Additional file 8). In summary, we show that miR-363-5p regulate two genes involved in extracellular matrix remodeling in EC. Importantly, we show that miR-363-5p regulate the angiocrine gene TIMP1 at post-transcriptional level.

#### miR-363-5p modulation affects EC:hematopoietic precursors communication

**Figure 6:**
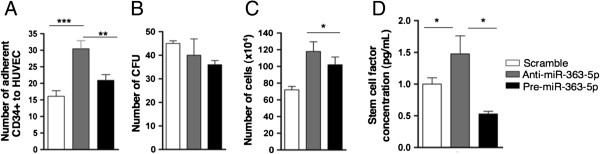
**Effect of miR-363-5p levels in the communication between endothelial cells:hematopoietic progenitor cells. (A)** Adhesion of CD34+ cells to HUVEC 24 h post-transfection with scramble control, anti-miR-363-5p or pre-miR-363-5p with or without the addition of recombinant stem cell factor (SCF). Error bars represent s.e.m. of adherent cells average by field (20x magnification) counted by light microscopy in three replicates. ** P ≤ 0.01, ***P ≤ 0.001 by Student’s t test. **(B)** Number of colony-forming units on methylcellulose of CD34+ cells after a 24 h exposure to transfected HUVECs. Total colony number was counted after 7 days on methylcellulose. **(C)** Number of HUVEC, 48 h post-transfection with scramble control, anti-miR-363-5p or pre-miR-363-5p. Data represent the average of cell counting (20x magnification) by light microscopy ± s.e.m. in three replicates. *P ≤ 0.05 by Student’s t test. **(D)** Detection of SCF levels by ELISA in HUVEC conditioned media 48 h post-transfection with scramble control, anti-miR-363-5p or pre-miR-363-5p. Data represent the average of the SCF concentration from three transfection experiments ± s.e.m. *P ≤ 0.05 by Student’s t test.

We exploited the possibility that miR-363-5p modulation, since it affected angiocrine factor expression on EC, might affect also their interaction with hematopoietic progenitor cells. To investigate the role of miR-363-5p in this process, we performed an adhesion assay where hematopoietic progenitor cells (CD34+) were placed in contact with EC having baseline, reduced or increased miR-363-5p levels. As shown in Figure 6, co-culture assays of EC and CD34+ cells showed that miR-363-5p reduction increased CD34+ cells adhesion to EC (Figure 6A), increased CD34+ proliferation (Figure 6B) but did not affect the colony-forming capacity of CD34+ cells on methylcellulose (CFUs) assays (a measure of the capacity of hematopoietic progenitors to differentiate into different hematopoietic lineages; Figure 6C). These data show that miR-363-5p modulation in EC promotes hematopoietic progenitors adhesion and proliferation. Next, we sought to identify the possible mechanisms modulated by miR-363-5p that could explain these observations. We observed that among the different angiocrine genes modulated by miR-363-5p in EC, kit ligand (also termed stem cell factor, SCF) was significantly induced by miR-363-5p reduction, as determined by qRT-PCR (Figure 3B) and also from cDNA microarrays data (data not shown). Although SCF is not predicted to be a direct target of miR-363-5p, its expression is modulated upon changes in miR-363-5p levels in EC. Considering the relevant role of SCF in the communication between EC and hematopoietic precursor cells, soluble SCF was quantified in the supernatant of EC transfected with anti-miR-363-5p, pre-miR-363-5p or scramble control. As shown in Figure 6D the reduction of miR-363-5p in EC resulted in an increase of soluble SCF, whereas increased miR-363-5p in EC lead to a significant decrease in soluble SCF release. Importantly, we performed a rescue experiment by adding recombinant SCF to our co-cultures. The expectation was that adding SCF might affect the CD34+ precursor cells and -at least partially- block their adhesion onto EC transfected with anti-miR.363-5p. As shown in Figure 6A, addition of SCF significantly reduced the number of adherent CD34+ to anti-miR-363-5p transfected EC. This was accompanied by a massive increase in non-adherent CD34+ cells in the co-culture supernatants (not shown). Together, these results suggest that miR-363-5p levels in EC affect the communication with CD34+ hematopoietic precursors through a mechanism that at least partially, involves the regulation of SCF expression and availability.

#### miR-363-5p modulation affects EC angiogenic properties

**Figure 7:**
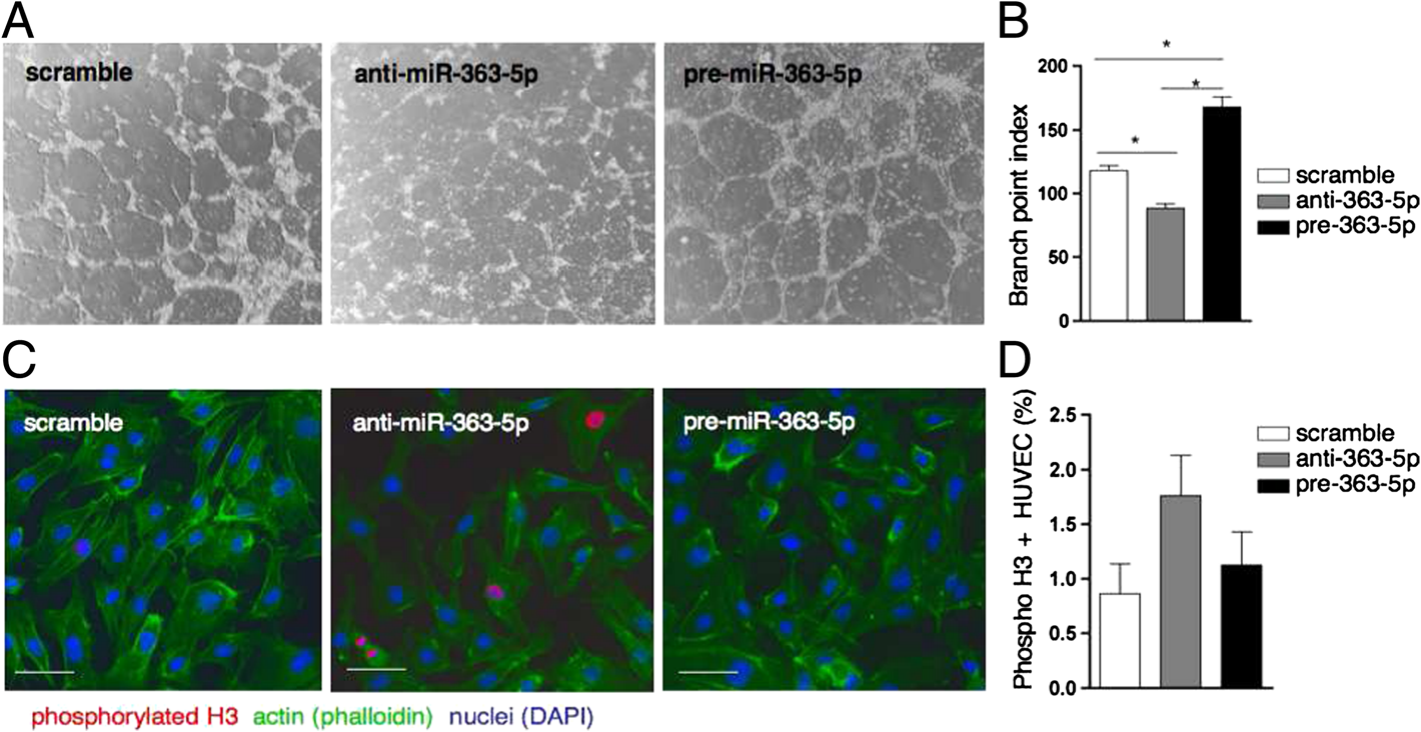
**Effect of miR-363-5p levels in EC properties. (A)** Tube formation assay to measure angiogenesis activity in HUVEC 48 h post-transfection with anti-miR-363-5p, pre-miR-363-5p and scramble control. Representative phase contrast (20X magnification) and **(B)** quantification by branch point index. Data are means ± s.e.m. of the two replicates from two independent experiments. * P ≤ 0.05 by Student’s test. **(C)** Proliferation assessed by positive staining for phosphorylated histone H3 (red) 48 h post-transfection with anti-miR-363-5p, pre-miR-363-5p and scramble control. Actin cytoskeleton was stained with phalloidin (green), nuclei, DAPI (blue). Scale bars, 10 μm. **(D)** Quantification represents the average ± s.e.m. of three independent transfection experiments.

Having shown that miR-363-5p reduction affected the expression of angiocrine factors, next we investigated whether miR-363-5p modulation affected also EC angiogenic properties. The increase of miR-363-5p levels promoted EC ability to form capillary-like structures on matrigel and conversely, the miR-363-5p reduction in EC affected severely their ability to form capillary-like structures (Figure 7A and B). In contrast, miR-363-5p reduction promoted EC proliferation, while miR-363-5p increase did not affect it (Figure 7C and D). Together, these data show that perturbation of miR-363-5p levels on EC affect endothelial angiogenic properties.

### Discussion

Similar to solid tumors, neo-vessel formation (angiogenesis) has been associated with disease progression in hematological cancers including leukemias and lymphomas [12-14]. This structural role of blood vessels in the BM is therefore linked to the needed increase in nutrients and oxygen to “feed” the expanding malignant cell clones. Nevertheless, recent evidence suggests BM vessels may play a more integrative role in the BM microenvironment, providing “instructive” or “angiocrine” cues to hematopoietic cells, this way maintaining BM homeostasis [3]. This dynamic interaction between endothelial cells of BM vessels and hematopoietic elements (immature, undifferentiated precursors and differentiated progeny) should be tightly regulated, maintaining the balance between mature and immature hematopoietic cells and contributing towards BM recovery when needed. Nevertheless, the molecular signals that regulate angiocrine factor production and BM function are largely unknown and were the subject of the present study.

miRNAs are key regulators of gene expression at post-transcriptional level and are implicated in a wide range of biological functions including cell proliferation, differentiation, apoptosis, among many others [15]. miRNAs deregulation is associated with several cancers [16]. The miRNA expression profiles show that the vast majority of the miRNAs is down-regulated in many cancers [17]. Interestingly, there has been a significant interest in the identification of miRNAs that selectively regulate EC function, namely during tumor angiogenesis. The term “angiomiRs” was coined a few years ago, to include the miRNAs that regulate particular EC functions [18]. Nevertheless, to our knowledge, a specific miRNA regulating angiogenic and angiocrine properties on EC was not reported. We reasoned that the molecular profiling of BM EC exposed to stress might reveal the genes and target pathways involved in the homeostatic function of EC in BM microenvironment. For this, we used a well-established approach to induce BM stress (which consisted of whole body sub-lethal irradiation), isolated the EC from irradiated or control BM and discovered a set of miRNAs that are induced on BMEC following whole body irradiation. The miRNA profiling of irradiated BMEC revealed a large number of differentially expressed miRNAs from the non-irradiated control. Subsequent validation of miRNA induction by irradiation *in vitro* allowed further mechanistic studies to be developed.

In detail, we identified one particular miRNA (miR-363-5p) that is induced by irradiation and selectively regulates EC properties, including the expression of angiocrine factors that are involved in the communication between BMEC and hematopoietic precursor cells. Interestingly, miR-363-5p previously named miR-363* is a miRNA generated from the upload of the miRNA* strand into RISC, which confirms the earlier observation that miRNA* are not always degraded and are active post-transcriptional gene regulators [19]. miR-363-5p was consistently induced by irradiation and was found to modulate the expression of angiocrine factors. miR-363-5p regulates (directly and indirectly) the expression and availability of well known angiocrine and hematopoietic factors (although their altered expression could, in fact occur as a result of a feedback response to miR-363-5p modulation per se). We showed by luciferase reporter assays that miR-363-5p regulates the expression of TIMP1 and THBS3 at post-transcriptional level. Variations in the expression of these targets at the protein level will be addressed in future studies in our Laboratory, particularly since THBS3 biological functions are largely unknown, and thus the effect of its regulation by miR-363-5p and the relevance for bone marrow homeostasis remains to be investigated.

As there were no reports in the literature about the pathways or genes that miR-363-5p could regulate and as target identification can be complex, we followed a transcriptome analysis upon forced modulation of the miR-363-5p levels. This strategy was previously reported [20] and was particularly useful to narrow-down the search of direct targets of a given miRNA. As miRNAs action depends on the miRNA-target stability, the strategy used, although allowed the identification of miRNA targets can result in the under-estimation of the targets identified, which can be dozens for a single miRNA [21]. Importantly, along with the targets directly regulated by miR-363-5p, its function may be enhanced by indirect mechanisms, as shown with the indirect regulation of kit ligand (stem cell factor). Kit ligand is essential for normal BM function and for BM recovery following irradiation [22]. In addition, TIMP1 is an inhibitor of matrix metalloproteinase function [23]. Earlier studies had shown the activation of MMP9 in the BM microenvironment tightly regulates the cleavage of kit ligand from a membrane bound to a soluble form, promoting BM recovery through hematopoietic precursors mobilization, differentiation and proliferation [24]. In the present report we show that EC with reduced miR-363-5p promote hematopoietic precursors adhesion and expansion, which is accompanied (and may be at least partially explained) by increased SCF production and release. The data presented here suggests that regulation of miR-363-5p expression on BMEC may regulate the *availability* of SCF in the BM microenvironment, highlighting the relevance of this particular miRNA in BM homeostasis.

### Conclusions

We provide evidence for the existence of an “angiomiR” induced by irradiation, the miR-363-5p, that regulates EC properties including the control of angiocrine factors production and release. Further studies implicating the importance of miR-363-5p in angiogenic or angiocrine situations are warranted.

### Methods

#### Mice irradiation and isolation of BMEC

All animal experiments were performed with the approval of the Instituto Gulbenkian de Ciência Animal Care Committee and Review Board. The mice were sub-lethally irradiated as previously published [25,26]; in the present study, FVB mice were used as subjects. miRNA profiling was performed in whole BM and in BMEC: the whole BM were extracted after irradiation as previously described and mononuclear cells were separated using Ficoll (Histopaque-1077). The BMEC were isolated using anti-CD31 fluorescein-conjugated Abs (1:100, Chemicon) and recovered using fluorescence-activated cell sorting (Modular Flow Cytometer, Beckman Coulter). Purity of recovered BMEC was ≥ 95%. Cells were centrifuged and the pellet was kept in TRIzol for further RNA extraction. Total BM from non-irradiated and irradiated mice was also used for RNA extraction as above.

#### RNA extraction, cDNA and qRT-PCR

Total RNA was extracted using TRIzol Reagent (Invitrogen™) according manufacturer instructions. mRNA and miRNAs were quantified according to the two protocols following described: for miRNA quantification, the cDNA was synthesized from 500 ng of total RNA using the NCode™ miRNA first-strand synthesis (Invitrogen™). miRNAs were quantified by quantitative RT-PCR (qRT-PCR) with SYBR Green (Invitrogen™) using a Universal qRT-PCR primer provided and primers to target specific miRNAs. Two μl of diluted cDNA (1:2) were used as template in 20 μl qRT-PCR reactions with 10 μM of each primer and 1x Platinum SYBR Green qRT-PCR Super Mix-UDG. The expression of U6 was used as endogenous control. miRNA levels were calculated using the comparative method 2^(−ΔΔCt) [27]. To perform the quantification of coding genes, the cDNA was synthesized from 1 μg of total RNA and random hexamers and Superscript II (Invitrogen) were used according to manufacturers instructions. Two μl of diluted cDNA (1:2) were used as template in 20 μl qRT-PCR reactions with 10 μM of each primer and SYBR Green (Applied Biosystems). The relative mRNA levels were normalized against 18S rRNA expression and calculated using the comparative method 2^(−ΔΔCt) [27]. All quantifications were performed with an ABI PRISM 7900HT Sequence Detection System (Applied Biosystems). Primers used in this study are available in Additional file 9. All reactions were run in triplicate.

#### miRNA profiling

Total RNA was isolated using TRIzol Reagent (Invitrogen). Quality and quantity of total RNA was analysed using the Agilent 2100 Bioanalyser (Agilent Technologies) and NanoDrop 2000. The miRNA profiling was performed using the miRCURY LNA (locked nucleic acid) V10.0 microarrays (Exiqon). The labeling was performed according to manufacturers recommendations using the miRCURY LNA microRNA Power labeling kit (Exiqon) from1 μg of total RNA (BMEC and whole BM). The microarrays used for miRNAs expression profiling comprised a total of 1154 probes from the miRBase Sequence Database version 8.0 (http://microrna.sanger.ac.uk) [28,29]. The hybridized microarrays were washed, dried and scanned using a dual-laser Agilent Technologies scanner. Scanned images were analyzed using Feature Extraction Software (Agilent Technologies), which converts scanner-generated images into quantitative log2 ratios. Labelling efficiency was evaluated by the signals from the control spike-in capture probes. Background correction and normalization was performed using the Local Nearest Neighbour algorithm Lowess (locally weighted scatterplot smoothing regression algorithm), which uses multiple local backgrounds in the neighbourhood of a given spot to serve as background signal for that feature. Expression values were presented as log2 ratio of red signal/green signal. Log2 ratio errors and associated p-values, which determine the probability that a log ratio is significantly different form zero, were also calculated. The final expression values of each miRNA correspond to the average of the quadruplicates spots within the slide. The expression values were submitted to Nudge algorithm to identify differentially expressed miRNAs. TIGR Multiple Experiment Viewer software package (MeV version 4.1; [30]) and Excel was used to perform data analysis and visualize the results.

#### Bioinformatic integration of mRNA and miRNA expression data

The miRNA target prediction was performed using databases available online: miRanda and miRBase (http://microrna.sanger.ac.uk; [28,29], TargetScan (http://www.targetscan.org; [31], DIANA microT (diana.pcbi.upenn.edu; [32] and PicTar (http://pictar.mdc-berlin.de; [33].

#### Cell culture, transfection and functional analyses of miRNAs

Endothelial cells (Human Umbilical Vein Endothelial Cells - HUVEC) were cultured in EBM-2 complete medium supplemented with 5% foetal bovine serum (FBS). Passages older than 6 were not used in the transfection experiments. The anti-miR-363-5p, pre-miR-363-5p and scramble control (Ambion) were electroporated using HUVEC at 70% confluency at final concentration of 50 nM according to the manufacturers protocol (Nucleofector V kit VCA-1001; Amaxa). After 12 h, the media was replaced with fresh media. Experiments were performed 24 h post-electroporation.

#### Endothelial in vitro tube formation assay

The HUVEC (100,000 cells per well) transfected with anti-miR-363-5p or scramble control were seeded on 24-well plate coated with 200 μl of Growth Factor Reduced Matrigel (BD Biosciences). Branches were quantified after a 16 h incubation at 37°C. Three biological replicates were performed for each condition. Photographs were captured at 20x magnification using an Olympus Microscope.

#### Immunofluorescence

The HUVEC were fixed 48 h post-transfection with PFA (2%) for 10 min at room temperature. Cells were washed twice in PBS 1x and incubated with PBS/0.1%BSA for 30 min at room temperature. Cells were stained with phalloidin (1 μg/ml) for 30 min at room temperature, washed and mounted with Vectashield containing DAPI. Preparations were examined using a fluorescence microscope (Axioplan Microscope, Zeiss).

#### PCR array

Expression profiles of angiogenesis-related genes were generated using PCR Arrays (SABiosciences) in accordance with the manufacturers recommendations in an ABI PRISM 7900HT Sequence Detection System (Applied Biosystems). The RNA of HUVEC transfected with anti-miR-363-5p or scramble control was extracted as above and 1 μg was treated with DNase followed by cDNA synthesis using RT^2^ First Strand kit (SABiosciences). cDNA samples were mixed with RT^2^ qRT-PCR master mix and distributed across the PCR array 96-well. Fold-change of each gene from anti-miR-363-5p to scramble control HUVEC was reported as Log10(2^(−ΔΔCt)). Fold-changes greater or less than 0 are indicated as up- or down-regulation, respectively.

#### CD34+ isolation and adhesion experiment

Mononuclear cells from human cord bloods were separated over Ficoll and CD34+ progenitor cells were further isolated using magnetic beads (Miltenyi Biotec) following manufacturers instructions. Purity of the CD34+ was assessed by flow cytometry. CD34+ progenitor cells (1000 cells) were added to resting HUVEC monolayers that were previously (24 h before) transfected with anti-miR-363-5p, pre-miR-363-5p or scramble control on 24-well plates. As a rescue experiment, to test whether CD34+ cells adhesion to HUVEC transfected with anti-miR-363-5p, pre-miR-363-5p or scramble control could be blocked by increasing the levels of SCF, recombinant human stem cell factor (Life Technologies) was added (10 ng/mL).

Adherent CD34+ cells onto HUVEC were counted manually using a phase-contrast microscope. Triplicates were used for each experimental condition.

#### Colony-forming units (CFU) assay

Cord blood-derived CD34+ cells isolated as described above were assessed for CFU frequency by culturing them in methylcellulose (R&D Technologies) according to manufacturers instructions. Cells were cultured in triplicate for seven days after which colonies were counted and morphologically analyzed.

#### Luciferase reporter assay

For the luciferase reporter experiments, the UTRs of putative miR-363-5p target genes were amplified and cloned in a luciferase reporter vector (pMIR-REPORT, Ambion) downstream the luciferase gene. Specifically, the wild-type UTRs having the miRNA binding(s) sites were amplified by PCR using primers having *Spe*I and *Hind*III restriction sites. The PCR products and the vector were then digested with *Spe*I and *Hind*III, cloned into pMIR-REPORT and transformed into *E. coli* (One-Shot, Invitrogen). All constructs were verified by sequencing. Resulting plasmids were co-transfected (1.5 μg) into HUVEC with anti-miR-363-5p or pre-miR-363-5p or scramble (50 nM) and pRL-SV40 vector (0.5 μg) (Promega), which contains a Renilla Luciferase gene to normalize transfection rates. Mutation of seed sequence of the miRNA-binding site was performed using the Site-Directed Mutagenesis Kit (Promega) and the mutated plasmids were used for transfection as above. Primers used are listed in the Additional file 9. Luciferase activity was assayed after 48 h using the Dual-Luciferase Reporter System (Promega) and was normalized against Renilla activity. Results represent Luciferase/Renilla ratios of three independent experiments.

#### ELISA for Stem Cell Factor (SCF)

The SCF levels were quantified in the supernatants of HUVEC transfected with anti-miR-363-5p, pre-miR-363-5p or scramble control, 48 h post-transfection by ELISA (R&D Systems), according manufacturers guidelines. The assay was performed twice and error bars represent s.e.m. of three transfection experiments.

#### Transcriptomic studies

Gene expression profiles were performed using microarrays (Affymetrix GeneChip Human Gene 1.0 ST array) using total RNA extracted with TRIzol from HUVEC transfected with anti-miR-363-5p, pre-miR-363-5p or scramble control. The level of miR-363-5p (upon forced reduction or increase) was confirmed by qRT-PCR before performing the microarrays. GeneChip Hybridization and scanning were performed at Instituto Gulbenkian de Ciência (http://www.igc.gulbenkian.pt).

#### Statistical analysis

Experiments were performed at least three times, unless indicated. Significance was performed using Student’s t test (p ≤ 0.05) or Anova, where indicated. Graphs show the standard error of the mean (s.e.m.) using Student’s t test. Single, double and triple asterisks indicate statistically significant differences: * p ≤ 0.05; ** p ≤ 0.01; *** p ≤ 0.001.

## Competing interests

The authors declare that they have no potential conflicts of interests.

## Authors’ contributions

AC carried out the conception and design, collection of the data, data analysis and interpretation and drafted the manuscript. JA participated in the identication of direct targets of miR-363-5p. CO partcipated in the in vivo experiments and isolation of BMEC. ALG carried out the CD34+ cells isolation and adesion assay. JV has been involved in the transcriptomic studies. SIA participated in the miRNA profilling. FC participated in the ELISA and data interpretation. FP performed the bioinformatic and statistical analysis of the microarrays. MR participated in the coordination and design of miRNA profilling experiments. SD conceived the study, participated in its design, and coordination, provide financial support, contributed with data analysis and interpretation, helped the manuscript writing. All authors read and approved the final manuscript.

## Supplementary Material

Additional file 1 **Validation of the miRNA expression data from microarrays by qRT-PCR.** BM non-irradiated control, whole BM and BMEC isolated according to the mouse BM dysfunction model were used from two independent experiments. Error bars represent s.e.m. of the mean expression. ** P ≤ 0.01 *** P ≤ 0.001 by Student’s t test. Click here for file

Additional flie 2 **miRNA expression of eight miRNAs recovered from the microarray data (from Figure** 1**) throughout the BM dysfuncton model.** Levels were quantified by qRT-PCR in whole bone marrow seven days after the first, second and third irradiations. Results confirm the great increase of miR-363-5p levels in the BM after the third irradiation. Click here for file

Additional file 3 **Levels of miR-363-5p detected by qRT-PCR in control non-irradiated HUVECs and BMEC.** Error bars represent s.e.m. of the mean expression. Click here for file

Additional file 4 **Silencing levels of miR-363-5p 48 h post-transfection with anti-miR-363-5p in EC.** Results in this graph are indicative as this quantification was routinely made for the experiments performed in this study to assess the efficiency of transfection. Error bars represent s.e.m. of the mean expression. *** P ≤ 0.001 by Student’s t test. Click here for file

Additional file 5 **Validation of angiocrine and angiogenic-related genes by qRT-PCR.** Five genes were selected (Notch4, interleukin8 (IL8), Jag1, KDR (VEGFR2) and Thrombospondin1 (THBS1)). Data represent the mean ± s.e.m. of the expression from two independent experiments. Click here for file

Additional file 6 **Genes modulated by miR-363-5p in endothelial cells.** Genes were selected from expression arrays (from Figure 4) and predicted to be direct targets (miRBase) regardless the fold change cutoff. Genes up-regulated upon miR-363-5p forced reduction (by transfection with anti-miR-363-5p) and repressed miR-363-5p levels increase (by transfection with pre-miR-363-5p). Validation by qRT-PCR and NCBI accession are shown. Highlighted in grey are genes validated by qRT-PCR. n.a.: not applicable, qRT-PCR not performed. Click here for file

Additional file 7 **Validation of transcriptomic data (Affymetrix microarrays) by qRT-PCR.** Ten genes were selected for validation based on pathways analyses using the Ingenuity software. Graph shows expression levels (log2 fold change) of selected genes in endothelial cells 48 h post-transfection with anti-miR-363-5p or pre-miR-363-5p normalized to scramble control. Error bars represent s.e.m. of normalized expression mean from three independent transfection experiments. Click here for file

Additional file 8 **miR-363-5p regulates thrombospondin-3 (THBS3).** (A) Schematic view of the THBS3 construct into pMIR-REPORT showing the predicted binding site in the 3’UTR. Sequence alignment of miR-363-5p and the 3’UTR THBS3 is shown. Nucleotide mutations achieved by site-directed mutagenesis are colored in red. (B) Renilla luciferase activity normalized to Firefly activity of HUVEC 48 h post-transfection with pre-miR-363-5p relative to scramble control. Relative luciferase activity of wild-type and mutated 3’UTR constructs are shown. Errors bars are s.e.m. from three independent transfection experiments. * P ≤ 0.05, *** P ≤ 0.001 by Student’s t test. Click here for file

Additional file 9 **Sequence of the primers and experimental application.** h, Human; m, Mouse. Sequence of all the miRNAs used in the study are also shown. Click here for file
