# Supplementary material for: miR-363-5p regulates endothelial cell properties and their communication with hematopoietic precursor cells
Source: J Hematol Oncol. 2013 Nov 21;6:87. doi: 10.1186/1756-8722-6-87 (PMC3874849; doi:10.1186/1756-8722-6-87)
Supplement: Additional file 1 — Validation of the miRNA expression data from microarrays by qRT-PCR. BM non-irradiated control, whole BM and BMEC isolated according to the mouse BM dysfunction model were used from two independent experiments. Error bars represent s.e.m. of the mean expression. ** P ≤ 0.01 *** P ≤ 0.001 by Student’s t test. [file 1756-8722-6-87-S1.pdf]

Additional file 1

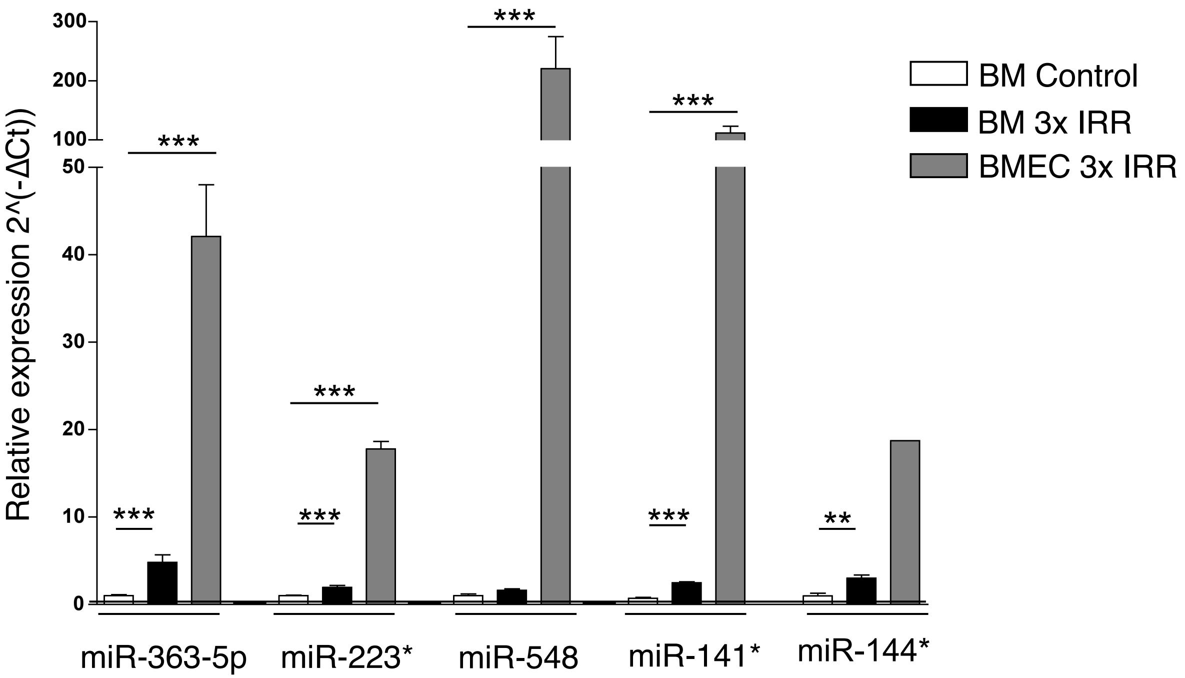

Additional file 1 - Validation of the miRNA expression data from microarrays by qRT-PCR. BM non-irradiated control, whole BM and BMEC isolated according to the mouse BM dysfunction model were used from two independent experiments. Error bars represent s.e.m. of the mean expression. ** P≤0.01 *** P≤0.001 by Student's t test.
