# Supplementary material for: miR-363-5p regulates endothelial cell properties and their communication with hematopoietic precursor cells
Source: J Hematol Oncol. 2013 Nov 21;6:87. doi: 10.1186/1756-8722-6-87 (PMC3874849; doi:10.1186/1756-8722-6-87)
Supplement: Additional flie 2 — miRNA expression of eight miRNAs recovered from the microarray data (from Figure 1) throughout the BM dysfuncton model. Levels were quantified by qRT-PCR in whole bone marrow seven days after the first, second and third irradiations. Results confirm the great increase of miR-363-5p levels in the BM after the third irradiation. [file 1756-8722-6-87-S2.pdf]

**Additional file 2**

**Additional file 2 - miRNA expression of eight miRNAs recovered from the microarray data (from Figure 1) throughout the BM dysfuncton model.** Levels were quantified by qRT-PCR in whole bone marrow seven days after the first, second and third irradiations. Results confirm the great increase of miR-363-5p levels in the BM after the third irradiation

| miRNA | Fold change compared to non-irradiated mice control after first, second and third irradiations | | |
|---|---|---|---|
| | 1x | 2x | 3 x |
| hsa-miR-363* | 0.9 | 1.56 | 6.57 |
| hsa-miR-548 | 0.99 | 0.91 | 2.03 |
| hsa-miR-223* | 0.68 | 0.92 | 1.06 |
| mmu-miR-136 | 0.72 | 0.61 | 0.88 |
| hsa-miR-451 | 1.83 | 1.60 | 0.58 |
| hsa-miR-141* | 1.85 | 0.76 | 0.29 |
| hsa-miR-144 | 0.35 | 0.13 | 0.01 |
| hsa-miR-144* | 0.89 | 1.18 | 0.01 |
