# Supplementary material for: miR-363-5p regulates endothelial cell properties and their communication with hematopoietic precursor cells
Source: J Hematol Oncol. 2013 Nov 21;6:87. doi: 10.1186/1756-8722-6-87 (PMC3874849; doi:10.1186/1756-8722-6-87)
Supplement: Additional file 3 — Levels of miR-363-5p detected by qRT-PCR in control non-irradiated HUVECs and BMEC. Error bars represent s.e.m. of the mean expression. [file 1756-8722-6-87-S3.pdf]

Additional file 3

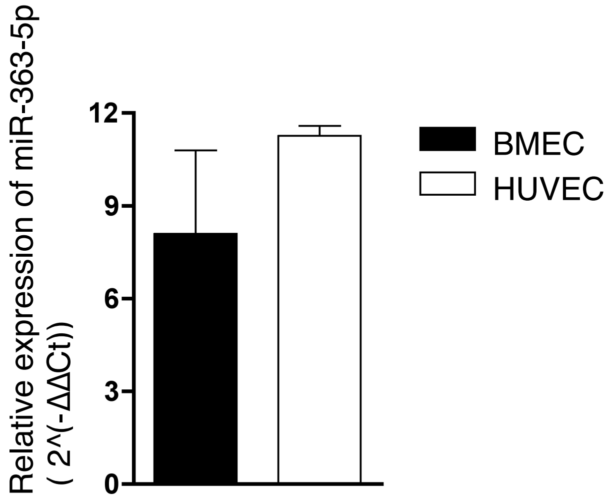

Additional file 3 - Levels of miR-363-5p detected by qRT-PCR in control non-irradiated HUVECs and BMEC. Error bars represent s.e.m. of the mean expression.
