# Supplementary material for: miR-363-5p regulates endothelial cell properties and their communication with hematopoietic precursor cells
Source: J Hematol Oncol. 2013 Nov 21;6:87. doi: 10.1186/1756-8722-6-87 (PMC3874849; doi:10.1186/1756-8722-6-87)
Supplement: Additional file 4 — Silencing levels of miR-363-5p 48 h post-transfection with anti-miR-363-5p in EC. Results in this graph are indicative as this quantification was routinely made for the experiments performed in this study to assess the efficiency of transfection. Error bars represent s.e.m. of the mean expression. *** P ≤ 0.001 by Student’s t test. [file 1756-8722-6-87-S4.pdf]

Additional file 4

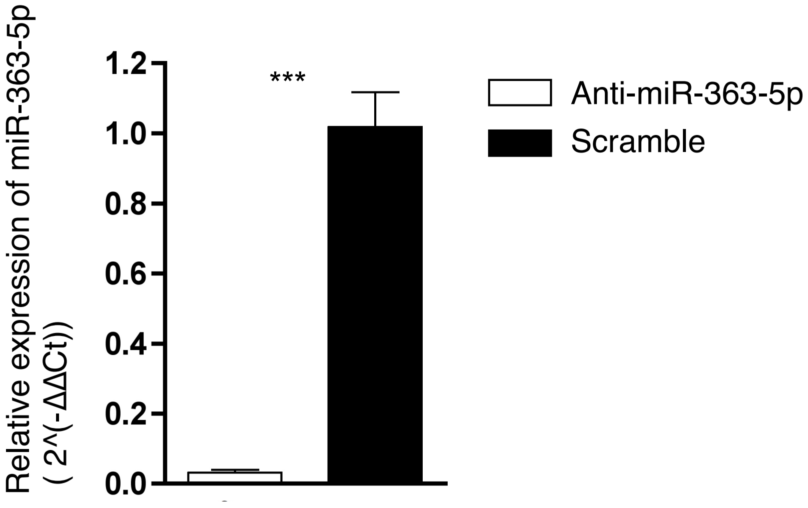

Additional file 4 - Silencing levels of miR-363-5p 48h post-transfection with anti-miR-363-5p in EC. Results in this graph are indicative as this quantification was routinely made for the experiments performed in this study to assess the efficiency of transfection. Error bars represent s.e.m. of the mean expression. *** P≤0.001 by Student's t test.
