# Supplementary material for: miR-363-5p regulates endothelial cell properties and their communication with hematopoietic precursor cells
Source: J Hematol Oncol. 2013 Nov 21;6:87. doi: 10.1186/1756-8722-6-87 (PMC3874849; doi:10.1186/1756-8722-6-87)
Supplement: Additional file 5 — Validation of angiocrine and angiogenic-related genes by qRT-PCR. Five genes were selected (Notch4, interleukin8 (IL8), Jag1, KDR (VEGFR2) and Thrombospondin1 (THBS1)). Data represent the mean ± s.e.m. of the expression from two independent experiments. [file 1756-8722-6-87-S5.pdf]

Additional file 5

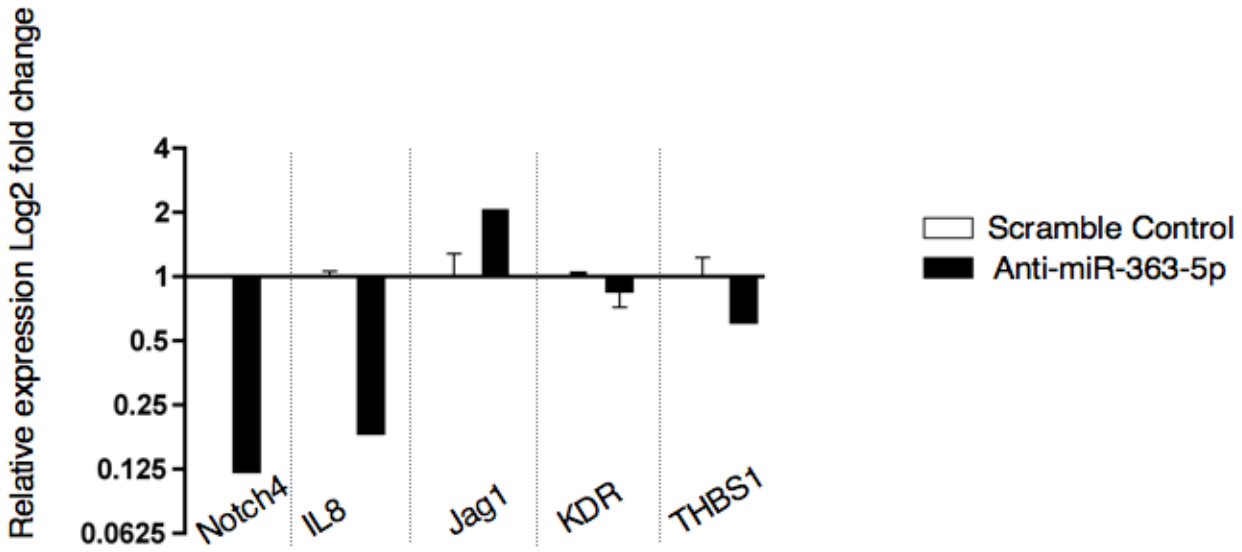

Additional file 5 - Validation of angiocrine and angiogenic-related genes by qRT-PCR. Five genes were selected (Notch4, interleukin8 (IL8), Jag1, KDR (VEGFR2) and Thrombospondin1 (THBS1)). Data represent the mean ± s.e.m. of the expression from two independent experiments.
