# Supplementary material for: miR-363-5p regulates endothelial cell properties and their communication with hematopoietic precursor cells
Source: J Hematol Oncol. 2013 Nov 21;6:87. doi: 10.1186/1756-8722-6-87 (PMC3874849; doi:10.1186/1756-8722-6-87)
Supplement: Additional file 6 — Genes modulated by miR-363-5p in endothelial cells. Genes were selected from expression arrays (from Figure 4) and predicted to be direct targets (miRBase) regardless the fold change cutoff. Genes up-regulated upon miR-363-5p forced reduction (by transfection with anti-miR-363-5p) and repressed miR-363-5p levels increase (by transfection with pre-miR-363-5p). Validation by qRT-PCR and NCBI accession are shown. Highlighted in grey are genes validated by qRT-PCR. n.a.: not applicable, qRT-PCR not performed. [file 1756-8722-6-87-S6.pdf]

Additional file 6

Additional file 6 - Genes modulated by miR-363-5p in endothelial cells. Genes were selected from gene expression arrays (from Figure 4) and predicted to be direct targets (miRBase) regardless the fold change cutoff. Genes up-regulated upon miR-363-5p forced reduction (by transfection with anti-miR-363-5p) and repressed miR-363-5p levels increase (by transfection with pre-miR-363-5p). Validation by qRT-PCR and NCBI accession are shown. Highlighted in grey are genes validated by qRT-PCR. n.a.: not applicable, qRT-PCR not performed

Gene	Description	NCBI acession	Fold enrichment Array 1	Fold down-regulation Array 2	Validation by qPCR
AMAC1L2	acyl-malonyl condensing enzyme 1-like 2	NM_054028	1.6	-1.84	NO
SLC25A45	solute carrier family 25	NM_182556	1.5	-1.6	n.a.
FCER1G	Fc fragment of IgE, high affinity I	NM_004106	1.3	-1.4	NO
IGFL3	IGF-like family member 3	NM_207393	1.3	-1.14	n.a.
IKBKG	inhibitor of kappa light polypeptide gene enhancer in B	NM_001099857	1.29	-1.18	YES
ACBD6	acyl-Coenzyme A binding domain containing 6	NM_032360	1.26	-1.37	n.a.
NANOG	Nanog homeobox	NM_024865	1.23	-1.29	n.a.
THBS3	thrombospondin 3	NM_007112	1.22	-1.4	YES
PPRC1	peroxisome proliferator-activated receptor gamma	NM_015062	1.2	-1.0	n.a.
BST1	bone marrow stromal cell1	NM_004334	1.19	-1.0	NO
ESAM	endothelial cell adhesion molecule	NM_138961	1.16	-1.09	NO
DOK3	docking protein 3	NM_024872	1.13	-1.2	n.a.
BAD	BCL2-antagonist of cell death	NM_004322	1.09	-1.11	NO
NPY	neuropeptide Y	NM_000905	1.06	-1.17	n.a.
MAP3K13	mitogen-activated protein kinase kinase kinase 13	NM_004721	1.06	-1.2	NO
SYT15	synaptotagmin XV	NM_031912	1.0	-1.3	n.a.
SELE	selectin E (endothelial adhesion molecule 1)	NM_000450	1.5	1.21	YES
TIMP1	TIMP metallopeptidase inhibitor 1	NM_003254	1.38	1.0	YES
