# Supplementary material for: miR-363-5p regulates endothelial cell properties and their communication with hematopoietic precursor cells
Source: J Hematol Oncol. 2013 Nov 21;6:87. doi: 10.1186/1756-8722-6-87 (PMC3874849; doi:10.1186/1756-8722-6-87)
Supplement: Additional file 7 — Validation of transcriptomic data (Affymetrix microarrays) by qRT-PCR. Ten genes were selected for validation based on pathways analyses using the Ingenuity software. Graph shows expression levels (log2 fold change) of selected genes in endothelial cells 48 h post-transfection with anti-miR-363-5p or pre-miR-363-5p normalized to scramble control. Error bars represent s.e.m. of normalized expression mean from three independent transfection experiments. [file 1756-8722-6-87-S7.pdf]

Additional file 7

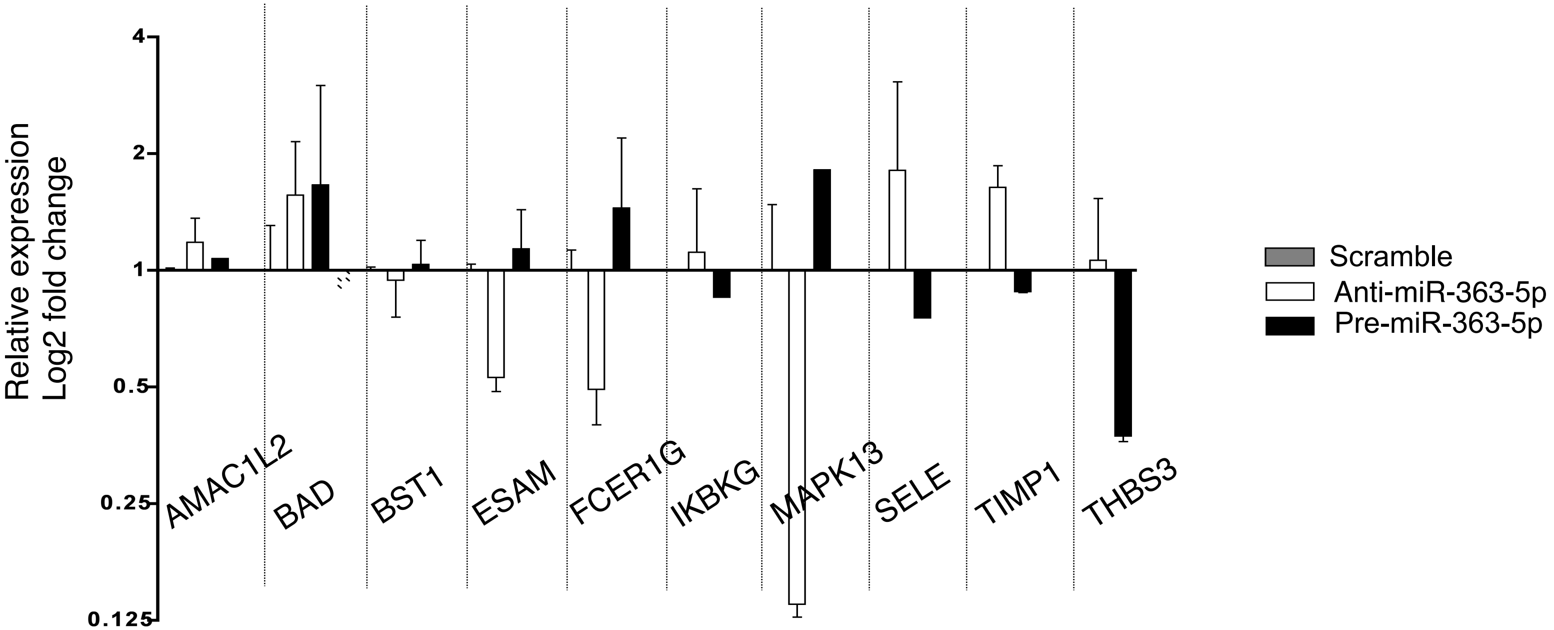

Additional file 7 - Validation of transcriptomic data (Affymetrix microarrays) by qRT-PCR. Ten genes were selected for validation based on pathways analyses using the Ingenuity software. Graph shows expression levels (log2 fold change) of selected genes in endothelial cells 48h post-transfection with anti-miR-363-5p or pre-miR-363-5p normalized to scramble control. Error bars represent s.e.m. of normalized expression mean from three independent transfection experiments .
