# Supplementary material for: miR-363-5p regulates endothelial cell properties and their communication with hematopoietic precursor cells
Source: J Hematol Oncol. 2013 Nov 21;6:87. doi: 10.1186/1756-8722-6-87 (PMC3874849; doi:10.1186/1756-8722-6-87)
Supplement: Additional file 8 — miR-363-5p regulates thrombospondin-3 (THBS3). (A) Schematic view of the THBS3 construct into pMIR-REPORT showing the predicted binding site in the 3’UTR. Sequence alignment of miR-363-5p and the 3’UTR THBS3 is shown. Nucleotide mutations achieved by site-directed mutagenesis are colored in red. (B) Renilla luciferase activity normalized to Firefly activity of HUVEC 48 h post-transfection with pre-miR-363-5p relative to scramble control. Relative luciferase activity of wild-type and mutated 3’UTR constructs are shown. Errors bars are s.e.m. from three independent transfection experiments. * P ≤ 0.05, *** P ≤ 0.001 by Student’s t test. [file 1756-8722-6-87-S8.pdf]

Additional file 8

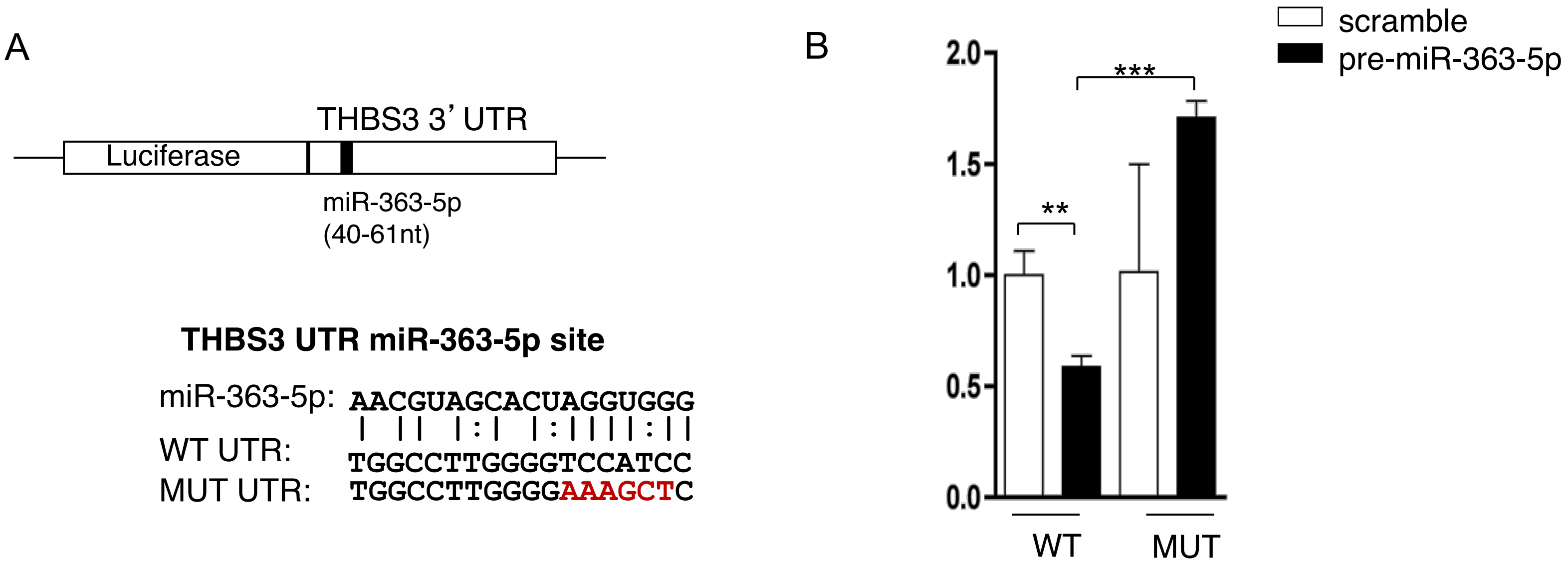

Additional file 8 - miR-363-5p regulates thrombospondin-3 (THBS3). (A) Schematic view of the THBS3 construct into pMIR-REPORT showing the predicted binding site in the 3'UTR. Sequence alignment of miR-363-5p and the 3'UTR THBS3 is shown. Nucleotide mutations achieved by site-directed mutagenesis are colored in red. (B) Renilla luciferase activity normalized to Firefly activity of HUVEC 48h post-transfection with pre-miR-363-5p relative to scramble control. Relative luciferase activity of wild-type and mutated 3'UTR constructs are shown. Errors bars are s.e.m. from three independent transfection experiments. * P≤0.05, *** P≤0.001 by Student's t test.
