# Supplementary material for: miR-363-5p regulates endothelial cell properties and their communication with hematopoietic precursor cells
Source: J Hematol Oncol. 2013 Nov 21;6:87. doi: 10.1186/1756-8722-6-87 (PMC3874849; doi:10.1186/1756-8722-6-87)
Supplement: Additional file 9 — Sequence of the primers and experimental application. h, Human; m, Mouse. Sequence of all the miRNAs used in the study are also shown. [file 1756-8722-6-87-S9.pdf]

Additional file 9

Sequence of the primers and experimental application. h, Human; m, Mouse.

Sequence of all the miRNAs used in the study are also shown.

Oligonucleotide	Sequence (5′ to 3′)	Purpose
h18S rRNA FWD	GCCCTATCAAACTTTCGATGGTAGT	normalization coding genes
h18S rRNA REV	CCGGAATCGAACCCTGATT	
mB-actin FWD	AGCCATGTACGTAGCCATCC	normalization coding genes
mB-actin FWD	CTCTCAGCTGTGGTGGTGAA	
hNotch4 FWD	TAGGGCTCCCAGCTCTC	validation of PCR arrays and/or angiocrine genes
hNotch4 REV	GGCAGGTGCCCCATT	
hIL8 FWD	CTGGCCGTGGCTCTCTTG	
hIL8 REV	CCTTGGCAAAACTGCACCTT	
hJAG1 FWD	CGGCTTTGCCATGTGCTT	
hJAG1 REV	TCTTCCTCCATCCCTCTGTC	
hTIMP1 FWD	TGTTGTTGCTGTGGCTGATAGC	
hTIMP1 REV	AATTGCAGAAGGCCGTCTGT	
hTHBS1 FWD	TGGAGCGGAAAGACCACTCT	
hTHBS1 REV	CCGCCTTGCCATTGGA	
hIGF1 FWD	GGTGGATGCTCTTCAGTTCGT	
hIGF1 REV	TACCCTGTGGGCTTGTTGAAA	
hSDF1 FWD	CAGATGCCCATGCCGATT	
hSDF1 REV	AGTTTGGAGTGTTGAGAATTTTGAGA	
hDLL1 FWD	ATGCCTTCGGCCACTTCAC	
hDLL1 REV	CACATCCAGGCAGGCAGAT	
hCXCR4 FWD	CAGTGGCCGACCTCCTCTT	
hCXCR4 REV	CAGTTTGCCACGGCATCA	
hDLL4 FWD	GTGGGTCAGAACTGGTTATTGGA	
hDLL4 REV	TGCAGATGACCCGGTAAGAGT	
hANG2 FWD	GCATTGAAGCTTAGTCCACCTGAGGAACTG	
hANG2 REV	CCGATACACTAGTCCTCGGGTTCATCTTTGC	
hBAMBI FWD	TGCCGTGCCCATTGCT	
hBAMBI REV	GCATCCTCAGGGCCAACA	
hANGPTL3 FWD	CACGAAACCAACTATACGCTACATCT	
hANGPTL3 REV	TGAAGTGTCCTTTTGCTTTGTGA	
hSCF FWD	GTGGATGACCTTGTGGAGTGCGT	
hSCF REV	GCCTGGGTTCTGGGCTCTTGA	
hAMAC1L2 FWD	CCCACCAAGCCAGATAGGAA	validation of transcriptomic analyses
hAMAC1L2 REV	GGGCTTCTGGTCTATCGTTCTCT	
hBAD FWD	CGAGTTTGTGGACTCCTTTAAGAAG	
hBAD REV	TCCCACCAGGACTGGAAGAC	
hBST1 FWD	TTACCGACCAGTGAAGCTCTTACA	
hBST1 REV	GCCGACTTTAAGGCACAGTCA	
hESAM FWD	CAGTGGGATCGGCAGCTT	
hESAM REV	AAAGACCCACGGATGACATCTAA	
hFCER1G FWD	GATGCCATCCTGTTTCTGTATGG	
hFCER1G REV	TTATAGCTGCCTTTCGCACTTG	
hIKBKG FWD	AGGACCCCGCAGACTATCAA	
hIKBKG REV	CTCGATGATGCTGGAGAGCTT	
hMAPK13 FWD	CTGTGCCCATGGACAACTCTAC	
hMAPK13 REV	TTCCTGTGGACCAGTCTACTAGCA	
hSELE FWD	TTCCCTTGGTAGCTGGACTTTC	
hSELE REV	TTTCCGAAGCCAGAGGAGAA	

hTHBS3 FWD	GGCGCACACACACAGTTCTC	
hTHBS3 REV	CTGCATGTTGGTCACCCAGTT	
TIMP1 FWD EXT	CCGATACACTAGTCTGGAACAGCCTGAGCTTAG	primers with restriction enzymes sites to clone into pMIR-REPORT
TIMP1 REV EXT	GCATTGAAGCTTCCGGAAGAAAGATGGGAGTG	
SELE FWD EXT	GCATTGAAGCTTTTCTGTTGCCTAGACTGGAGTG	
SELE REV EXT	CCGATACACTAGTGAACCAGACTGCCAGAGTTTG	
IKBKG FWD EXT	GCATTGAAGCTTGCTTTCCTCTCCCGCCTGCCTAGCC	
IKBKG REV EXT	CCGATACACTAGTCACCCGTGTGCATGGTAAG	
THBS3 FWD EXT	GCATTGAAGCTTGGCCCACCAGATTCAGAATTCAG	
THBS3 REV EXT	CCGATACACTAGTTTTGGGAGCCAGAGAAGG	
TIMP1 MUT1 FWD	TCGTGTTTCCCTGTTTAAGGTGGCCCCTGCAAACTG	primers for site-directed mutagenesis
TIMP1 MUT1 REV	TTAAACAGGGAAACACGACACGATTCCTCACAGCCAACAG	
TIMP1 MUT2 FWD	AGCTGAAGCCTGCACAGTGTGGTGGCTGTTCCCACTC	
TIMP1 MUT2 REV	ACACTGTGCAGGCTTCAGCTTCCACTC	
THBS3 MUT FWD	TTAGACCCTTTGGCCTTGGGGTCCATCCTGGAGACCCTGAG	
THBS3 MUT REV	CCCCAAGGCCAAAGGGTCTAAAATTCTGAATTCTGAATC	
U6 FWD	GTGCTCGCTTCGGCAGCACATATAC	normalization of miRNA levels
U6 REV	AAAAATATGGAACGCTTCACGAATTTG	
hsa-miR-363-5p	CGGGTGGATCACGATGCAATTT	miRNAs quantified by qRT-PCR
hsa-miR-223-5p	CGTGTATTTGACAAGCTGAGTT	
hsa-miR-548-3p	CAAAAACCACAGTTTCTTTTGC	
hsa-miR-141-5p	CATCTTCCAGTACAGTGTTGGA	
hsa-miR-144-5p	GGATATCATCATATACTGTAAG	
hsa-miR-92a-2-3p	TATTGCACTTGTCCCGGCCTGT	
hsa-miR-18b-5p	TAAGGTGCATCTAGTGCAGTTAG	
hsa-miR-20b-5p	CAAAGTGCTCATAGTGCAGGTAG	
hsa-miR-106a-5p	AAAAGTGCTTACAGTGCAGGTAG	
hsa-miR-19b-3p	TGTGCAAATCCATGCAAAACTGA	
pre-miR-363-5p FWD	GCAGCAACTAGAAACG	
pre-miR-363-5p REV	GCACTCATGCCCATTCATCC	
